# The Neuroimaging Role of Modified Electroconvulsive Therapy in the Major Depressive Disorder: Effectiveness in First-Episode Antipsychotic-Naive Major Depressive Disorder Patients

**DOI:** 10.1155/2024/9211145

**Published:** 2024-02-12

**Authors:** Yi Zhong, Jianfeng Li, Haitao Li, Mingzhe Li, Yanaohai Lyu, Minghu Cui, Yujun Gao

**Affiliations:** ^1^Department of Psychiatry, Renmin Hospital of Wuhan University, Wuhan 430000, Hubei, China; ^2^Peking University Sixth Hospital, Peking University Institute of Mental Health, NHC Key Laboratory of Mental Health (Peking University), National Clinical Research Center for Mental Disorders (Peking University Sixth Hospital), Peking University, Beijing 100191, China; ^3^Yi Zheng Hospital, Drum Tower Hospital Group of Nanjing, Nanjing, Jiangsu, China; ^4^Affiliated Shuyang Hospital of Nanjing University of Chinese Medicine, Suqian 223600, Jiangsu, China; ^5^Peking-Tsinghua Center for Life Sciences and PKU-IDG/McGovern Institute for Brain Research, Peking University, Beijing, China; ^6^Department of Social and Behavioral Sciences, City University of Hong Kong, HKSAR, China; ^7^Department of Psychiatry, Binzhou Medical University Hospital, Binzhou 256600, Shandong, China; ^8^Clinical and Translational Sciences (CaTS) Lab, The Douglas Research Centre, McGill University, Montréal, Québec, Canada

## Abstract

**Objectives:**

It is a high risk for adolescents with first-episode major depressive disorder (MDD) to commit suicide. However, few studies reported the effect of modified electroconvulsive therapy (MECT) in first-episode antipsychotic-naive MDD adolescents.

**Methods:**

The study explores the alternations of regional homogeneity of modified electroconvulsive therapy to treat the first-episode antipsychotic-naive major depressive disorder. 72 first-episode MDD patients were recruited from Tianyou Hospital Affiliated to Wuhan University of Science and Technology from October 2017 to May 2020, and 65 of 72 completed the trial.

**Results:**

Before MECT treatment, ReHo values of the bilateral cerebellum and left cuneus were higher, and ReHo value of left postcentral and supramarginal gyrus was lower in MDD patients compared to healthy subjects (HS). After treatment, the MDD patients have higher ReHo values of the right insula and postcentral gyrus, while left fusiform gyrus were lower than the pretreatment. Compared to the HS, the ReHo values of left lingual gyrus, right calcarine cortex, and right mid occipital thalamus were higher in the posttreatment. In the posttreatment, left calcarine cortex and right cerebrum were lower than in healthy subjects.

**Conclusions:**

The study confirmed that MECT improves psychotic symptoms in patients with first-episode antipsychotic-naive MDD. These results further contributed to a more tailored treatment approach to MDD from the pathophysiological and neuroimaging views.

## 1. Introduction

Major depressive disorder (MDD) is a widely distributed disease characterized by depressed mood, vegetative symptoms, diminished interest or pleasure in daily activities, and impaired cognitive function, such as disturbed sleep or appetite [1]. MDD is a complex mental health condition that affects individuals across all age groups, socioeconomic backgrounds, and cultures [2]. Nowadays, more than 264 million people are affected by MDD, which is associated with high costs to the global community [3]. Compared with men, MDD occurs about twice as often in women and affects about 6% of the adult population worldwide [4, 5]. According to the data publicized by the World Health Organization, MDD is the second leading cause of disability measured by years lived with disability, with global prevalence exceeding 4% [6–8]. While the exact causes of MDD are not fully understood, extensive research suggests that its introduction involves a combination of genetic predisposition, environmental influences, and alterations in neurobiological pathways [9]. Genetic factors play a substantial role in the development of MDD. Family and twin studies have shown that individuals with a family history of depression are at a higher risk of developing the disorder themselves [10, 11]. Specific genes and genetic variations related to neurotransmitter regulation, neuroplasticity, and stress response have been implicated in MDD's introduction and progression [12, 13]. However, it is important to note that genetics alone do not determine the onset of MDD, and environmental factors also play a significant role [14]. Environmental factors, such as early-life adversity, trauma, chronic stress, and interpersonal conflicts, can contribute to the introduction of MDD [15, 16]. These factors can disrupt neurodevelopmental processes, alter stress response systems, and increase vulnerability to depression [17]. Social determinants of health, including socioeconomic status, access to healthcare, and social support networks, also influence the likelihood of developing MDD [18]. Understanding these factors is crucial for identifying at-risk individuals and implementing preventive measures.

Further, for MDD, only 27% of patients remit after an initial trial, and first-line pharmacological treatments are inadequate [19]. Fortunately, modified electroconvulsive therapy (MECT) provides a highly effective treatment which can be used for depression induced under general anaesthesia. In the therapy, a generalized tonic-clonic seizure occurs by direct current stimulation through the scalp [20]. Several long-term follow-up studies have confirmed that patients receiving MECT have reduced mortality of all causes compared to the control group of non-ECT patients [21–23]. Several studies have also shown the antidepressant effect on MECT [24, 25]. There is no pharmacological treatment, or otherwise, that has equated with MECT in speed or likelihood of remission of MDD [23, 26].

However, it is unclear exactly how the treatment affects the brain, and approximately only half of MDD patients remitted when receiving MECT [21]. Neurobiological mechanisms play a vital role in MDD's introduction and manifestation. Resting-state functional magnetic resonance imaging (rs-fMRI) is an influential tool to detect spontaneous activity in the brain which focuses on low-frequency fluctuations [27, 28]. It has been disclosed that characteristics of resting state provide valuable insights into a disease's progression, contributing to enhancing knowledge of diseases [29, 30]. MDD patients showed increased functional connectivity in the left medial frontal cortex/occipital thalamus but a reduction in the bilateral precuneus, the left posterior parietal thalamus, and the posterior cingulate cortex. From the data of 25 Chinese research groups on neuroimaging of 1,300 depressed patients and 1,128 healthy controls, Yan et al. found that default mode network functional connectivity (DMNFC) is decreased in depressed patients, especially in patients with recurrent depression, which suggests that for understanding depression pathophysiology as well as identifying effective therapies, DMNFC remains a prime target [8]. Dysregulation in brain circuits involving areas responsible for mood regulation, such as the prefrontal cortex, amygdala, and hippocampus, has been observed in individuals with MDD [31–35].

Regional homogeneity (ReHo) is deployed to characterize the brain neural activity [36]. According to previous research, abnormal ReHo indicates local functioning imbalances or a decompensation reaction affecting the whole brain [37, 38]. ReHo is recommended as a potential biomarker for neuroimaging to trace functional homogeneity changes and gain insight into the neurophysiology of brain disorders [39]. The analysis of ReHo is incrementally utilized to measure functional synchronization in brain region presently. Then, it eventually reveals the mechanisms of psychiatric and neurological diseases, including depression [40], schizophrenia [41], and Alzheimer [42].

However, few studies reported the effect of modified electroconvulsive therapy (MECT) in first-episode antipsychotic-naive MDD adolescents. The study explores the resting-state regional homogeneity of resting-state brain abnormalities of MECT in treating the first-episode antipsychotic-naive major depressive disorder in adolescents. In our study, the primary aim is to explore the unique neuropathological mechanisms in MECT and identify whether an interaction between changes of MECT-induced brain connectome and clinical improvement emerges in first-episode MDD patients. To achieve the purpose, we compared resting-state fMRI ReHo map between first-episode antipsychotic-naive MDD patients with and without MECT in a cohort of 72 participants, including 36 first-episode antipsychotic-naive MDD and 36 healthy subjects. Of the 72 subjects, 65 completed the trial. Eventually, we analyzed the effect of MECT in the treatment of first-episode antipsychotic-naive MDD.

## 2. Materials and Methods

### 2.1. Participants

Seventy-two subjects were recruited from Tianyou Hospital Affiliated to Wuhan University of Science and Technology consecutively between January 2018 and January 2022. All patients were diagnosed with MDD per the criteria in the fifth version of the Diagnostic and Statistical Manual of Mental Disorders (DSM-V). Thirty-six participants who met inclusion criteria showed a 17­Item Hamilton Rating Scale for Depression (HRSD­17) [43] score of at least 17 currently. They showed no previous diagnosis and treatment for depression based on a neuropsychiatric interview confirmed by two psychiatric physicians independently in outpatient service. We set the scale assessment and scan points at the baseline time (1 day before) and after eight MECT sessions or all sessions if more than eight sessions (1 day after). In addition, to prevent the transformation from MDD to bipolar disorder, suicide, and other severe diseases, we interviewed the participants at regular intervals and excluded the patients who did not meet the inclusion criteria. During the process, the patients who needed antidepressants were also excluded from the participants to avoid interfering with the efficacy in clinical symptoms and brain activity.

Inclusion criteria for the first-episode MDD patient were as follows: (1) aged 18–30 years, Han ethnicity, and showed a willingness to participate in the research; (2) met the DSM-V criteria for MDD and HRSD-17 score ≥ 17; (3) active to undertake MRI scanning; and (4) committed to taking part in the whole procedure. The exclusion criteria for MDD patients were as follows: (1) met the criteria for schizophrenia, obsessive-compulsive disorder, bipolar disorder, or other psychotic disorders; (2) substance abuse in the past 3 months; (3) pregnancy or suckling; (4) personality disorder; (5) a history of loss of consciousness including neurological illness; (6) requiring medications or hospitalization; (7) claustrophobia or other contraindications; (8) left hand; and (9) injury to self or others.

Thirty-six healthy right-handed volunteers, age-, education-, and gender-matched to the MCI patients, who had no history and family history of psychiatric disorders and substance abuse and have not been treated with antipsychotics, were recruited through advertisements from the community via outpatient service or an online platform. Healthy subjects (HS) were screened using the structured interview from DSM-V to avoid the absence of psychiatric or neurologic illness at the medical center of Tianyou Hospital Affiliated to Wuhan University of Science and Technology. The research ethics board granted ethics approvals (wkd20191201). All participants or their guardian provided written consent before the study.

### 2.2. Electroconvulsive Therapy

A dose of 50 mg of fluvoxamine was initially given twice a day, and the dose was adjusted within two weeks depending on the condition and tolerance of the patients, with a maximum dose of 300 mg each day. Atropine 0.5 mg and etomidate fat emulsion 0.3 mg/kg were injected intravenously before MECT. In the next step, 0.2% succinylcholine (1.0–1.5 mg/kg) was injected intravenously, and a mask airbag was used for artificial respiration. An electroconvulsive device (Thymatron DGx, Somatics LLC, Lake Bluff, IL, USA) was used for bilateral electroconvulsive treatment of the forehead after fasciculations of the limbs. The intensity was administrated on age (2/3 of age in patient) [44, 45]. In the subsequent MECT sessions, the output dosage was adjusted according to the previous session's convulsive seizure level. The main ECT parameters were the following: maximum charge (504 mC), maximum duration (8 s), output current (0.9 A), pulse width (1 ms), and frequency (10–70 Hz) [44, 46]. Three courses of ECT were given consecutively on successive days, and the remaining courses were taken every two days with a weekend break in between. After eight sessions, sessions were continued if the patients did not achieve depressive symptom relief sufficiently, which was determined by the psychiatric physician. Physiological monitoring included electrocardiography (ECG) and blood pressure to monitor the physical condition. MRI and ECG were carried out to exclude severe somatic pathology [47].

### 2.3. Image Acquisition

All participants were scanned using an Achieva scanner (3.0T, Philips, Amsterdam, the Netherlands), with eyes closed and heads kept stable using a foam padding. After scanning, the participants were asked whether they fell asleep during the process. A sequence of EPI was used to collect images: repetition time: 2000 ms, echo time: 30 ms, flip angle: 90°, field of view: 220 × 220 mm^2^, matrix: 64 × 64, slice thickness: 5 mm, and number of slices: 31 [48]. 200 volumes were obtained, scanning time is 400 s, and then, 3D T1-weighted images were collected (repetition time: 8.35 ms, flip angle: 12°, echo time: 3.27 ms, field of view: 240 × 240 mm^2^, matrix: 256 × 256, and slice thickness: 1 mm), and the sagittal slice is 156.

### 2.4. Data Preprocessing

We processed imaging data from resting-state fMRI in DPABI [49] and discarded the first ten volumes of the functional resting-state images to reduce the influence of participants' adaption and initial instability signals. Slice timing and head motion correction were residual time point sequences. We excluded the imaging data of participants whose maximum displacement was more than 2 mm in *x*, *y*, or *z* axis, or 2° of maximum rotation [50]. The structural brain image (T1) was first aligned with the individual's functional (EPI) image. The structural T1 image was broken down into three main components: gray matter, white matter, and cerebrospinal fluid. After acquiring registration parameters from the initial alignment, the individual functional (EPI) image was then spatially normalized to a standardized brain template, known as the Montreal Neurological Institute (MNI) space. The voxel size of the images was resampled to a resolution of 3 × 3 × 3 mm^3^. During the normalization process of the functional images, any signal changes due to head motion, white matter activity, and cerebrospinal fluid activity were accounted for and removed. The processed image was then smoothed using a Gaussian kernel with a full width at half maximum (FWHM) of 6 mm. Finally, the data underwent a band-pass filtering process within the frequency range of 0.01 to 0.08 Hz. This helps in retaining the frequencies of interest while eliminating unwanted noise. Additionally, the data was linearly detrended to remove any low-frequency drifts that could distort the analysis [51].

### 2.5. ReHo Analysis

This method calculated Kendall's coefficient of concordance (KCC) in a given voxel's time series and those of its neighboring 26 voxels to produce an individual ReHo map. ReHo maps were divided by global means in order to reduce the influence of individual variation. The voxel-wise KCC was calculated to generate ReHo maps in a mask of gray matter. The mask was utilized to remove nonbrain tissues and background of maps from MNI template [52]. ReHo maps were prepared using BRANT software [53].

### 2.6. Statistical Analysis

Independent *t*-tests were used to analyze continuous variables with a normal distribution while nonnormally distributed data was analyzed using Mann–Whitney *U* statistics. The chi-square (*χ*^2^) test was used to compare the categorical variables, for example, the gender. Paired *t*-test was used to analyze the differences in HRSD-17 and BSSI at baseline and after treatment. The theory of Gaussian random fields (GRF) was utilized to adjust for multiple comparisons, leveraging the REST_V1.8 tool, with a threshold set at *p* < 0.01 for both individual voxel and cluster significance. The statistical analysis was implemented by SPSS 26.0. Analyses of neuroimaging data were conducted using DPABI and SPM12 [49]. A *p* value of < 0.05 was considered significant statistically.

## 3. Results

### 3.1. Demographic Data and Scales

Thirty-six first-episode MDD patients and thirty-six healthy subjects participated in the research. Three MDD patients discontinued treatment, and three MDD patients and one healthy subject were noncompliance. Eventually, thirty MDD patients and thirty-five healthy subjects were included. There were no significant differences in age (*p* = 0.415) and sex (*p* = 0.670). The details of data and statistical results are listed in Table 1. As is shown in Table 1, there was a significant difference in pre- and posttreatment scores of HRSD-17 (28.84 ± 6.017 vs. 13.58 ± 8.804, *p* < 0.001) and BSSI (21.77 ± 5.920 vs. 8.03 ± 6.834, *p* < 0.001).

### 3.2. ReHo Differences between Pre- and Posttreatment

**Figure 1:**
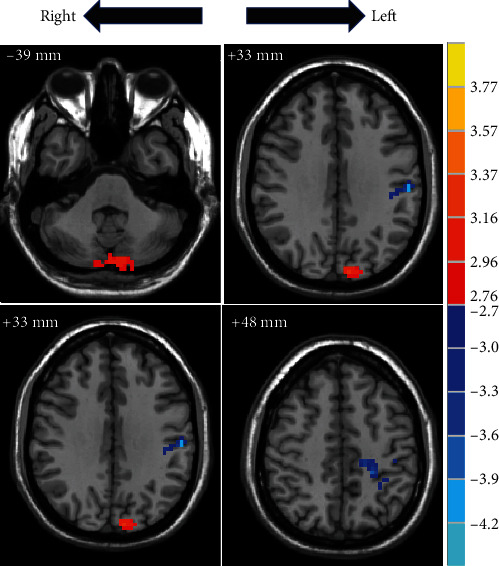
The difference in brain region between pretreatment and healthy subject. Two sample *t*-maps showed a significant ReHo difference between the pretreatment and healthy subjects. The red colour bar denotes relatively higher ReHo values in the pretreatment. The blue colour bar indicates relatively lower pretreatment values compared to healthy subjects. Brain region labels: bilateral cerebellum (MNI: *x*, *y*, *z* = 3, −87, −36, *t* = 3.2722), left cuneus (MNI: *x*, *y*, *z* = -9, -87, 36, *t* =3.6084), and left postcentral and left supramarginal gyrus (MNI: *x*, *y*, *z* = −57, −21, 33, *t* = −3.7236; MNI: *x*, *y*, *z* = −24, −36, 48, *t* = −3.4174).

The results revealed that ReHo values in the bilateral cerebellum (MNI: *x*, *y*, *z* = 3, −87, −36, *t* = 3.2722) and left cuneus (MNI: *x*, *y*, *z* = −9, −87, 36, *t* = 3.6084) were higher in the pretreatment than those in healthy subjects. The ReHo value of left postcentral and supramarginal gyrus (MNI: *x*, *y*, *z* = −57, −21, 33, *t* = −3.7236; MNI: *x*, *y*, *z* = −24, −36, 48, *t* = −3.4174) was lower in the pretreatment compared to those in healthy subjects (Figure 1).

**Figure 2:**
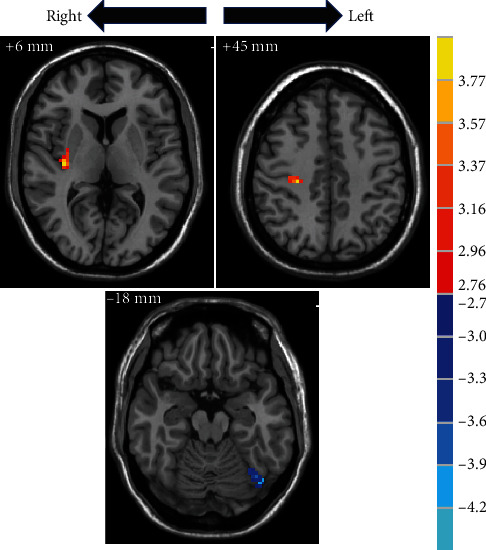
The difference in brain region between posttreatment and pretreatment. The red colour bar denotes relatively higher ReHo values posttreatment. The blue colour bar indicates relatively lower posttreatment values than pretreatment. Brain region labels: right insula (MNI: *x*, *y*, *z* = 36, −18, 6, *t* = 3.9763), right postcentral gyrus (MNI: *x*, *y*, *z* = 27, −24, 45, *t* = 3.7857), and left fusiform gyrus (MNI: *x*, *y*, *z* = −42, −72, −18, *t* = −4.0763).

Compared to the pretreatment, the ReHo values of right insula (MNI: *x*, *y*, *z* = 36, −18, 6, *t* = 3.9763) and right postcentral gyrus (MNI: *x*, *y*, *z* = 27, −24, 45, *t* = 3.7857) were higher and left fusiform gyrus (MNI: *x*, *y*, *z* = −42, −72, −18, *t* = −4.0763) was lower in the posttreatment (Figure 2).

**Figure 3:**
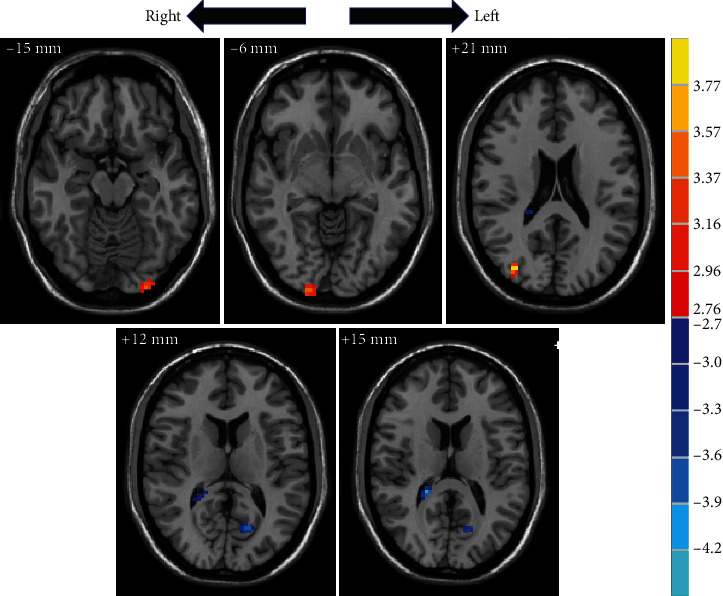
The difference in brain region between posttreatment and healthy subject. The red colour bar denotes relatively higher ReHo values posttreatment. The blue colour bar indicates relatively lower posttreatment values than pretreatment. Brain region labels: left lingual gyrus (MNI: *x*, *y*, *z* = −42, −72, −18, *t* = −4.0763), right calcarine cortex (MNI: *x*, *y*, *z* = 18, −102, −3, *t* = 3.4308), right mid occipital thalamus (MNI: *x*, *y*, *z* = 33, −84, 24, *t* = 3.9381), left calcarine cortex (MNI: *x*, *y*, *z* = −15, −69, 12, *t* = −3.3871), and right cerebrum (MNI: *x*, *y*, *z* = 21, −39, 18, *t* = −4.0722).

Compared to the healthy subjects, the values of left lingual gyrus (MNI: *x*, *y*, *z* = −42, −72, −18, *t* = −4.0763), right calcarine cortex (MNI: *x*, *y*, *z* = 18, −102, −3, *t* = 3.4308), and right mid occipital thalamus (MNI: *x*, *y*, *z* = 33, −84, 24, *t* = 3.9381) were higher in the posttreatment (Figure 3). Left calcarine cortex (MNI: *x*, *y*, *z* = −15, −69, 12, *t* = −3.3871) and right cerebrum (MNI: *x*, *y*, *z* = 21, −39, 18, *t* = −4.0722) were lower in the posttreatment than those in healthy subjects (Table 2).

## 4. Discussion

MDD is characterized by persistent feelings of sadness, loss of interest or pleasure, and a variety of accompanying symptoms that significantly impact daily functioning [2]. At global level, mental disorders are the seventh most common cause of disability-adjusted life years (DALYs) in 2019, compared to the 13th most common cause in 1990. Among all mental disorders, depressive disorders (37.3%) result in the maximum proportion of mental disorder DALYs according to the Global Burden of Disease Study (GBD) 2019 [54]. According to the DALY report, depression was ranked 13th out of the 25 leading causes and second in years lived with disability (YLDs) at the disorder level in GBD 2019. In terms of psychiatric disorders, depressive disorders ranked the highest in all age groups, from the 0- to 14-year age category [55]. In addition, MDD also lead to an increased risk of diabetes mellitus, heart disease, and stroke, thereby further increasing its disease burden [56].

Furthermore, MDD is also a risk factor of death caused by suicide. According to the WHO, up to 50% of the 700,000 suicides annually occur in a depressive episode worldwide [57]. Although the first-episode MDD patients' treatment has been confirmed to benefit from several antidepressants, the pharmacological antidepressant effects were delayed [26]. It may require 6 to 12 weeks to show optimal improvement after administration, while some patients, especially those exposed to acutely suicidal intent, need a rapid response [58]. Additionally, several difficulties were associated with the delayed onset effect of MDD treatment, such as reduced adherence to treatment, distress from patient and family, increased risk of suicidality, and economic burden [55, 59]. Those first-episode MDD patients need more treatment options associated with rapid-onset antidepressant effects to achieve early improvement.

As is known, electrode placement and pulse parameter alterations have significantly reduced the severity of cognitive side effects from ECT. Martin et al. reported that visual retrograde memory side effects were significantly associated with higher E-fields in regions of interest [60]. It was found that ultrabrief (UB: 0.3 ms) significantly enhanced visual retrograde memory retention and recognition compared to brief pulse (BP: 1.0 ms) across [60]. MECT has proved its efficacy in the treatment of MDD patients. Additionally, a previous study showed that MECT released symptoms of MDD patients significantly [21]. Chen et al. reported that a significant improvement was observed in the HAMD17 scores of depressed patients after the second MECT session in comparison to the baseline score [61]. However, MECT can aggravate poor working memory in depressed patients, though it may improve with depression relief [61]. A high MECT dosage and obesity may be associated with persistent deficits [62, 63].

In the study, we detected the changes in the signals and sought the region's differences between pre- and posttreatment. The result is that the scores of HRSD-17 and BSSI significantly decreased evidently after MECT. It means MECT can improve depressive disorders in MDD patients. To investigate the neuromechanism in MECT, resting-state magnetic resonance imaging, one of the essential tools for exploring mental disorders, was taken to explain the phenomena. ReHo maps were generated for not only patients but also healthy subjects.

To the best of our knowledge, the study is the first fMRI study to detect changes in brain region during treatment of antipsychotic-naive first-episode MDD patients by MECT with the method of ReHo. As is shown in the results, ReHo values in the left cuneus and bilateral cerebellum were higher in the pretreatment than those in healthy subjects. The ReHo value of left postcentral and supramarginal gyrus was lower in the pretreatment than that in healthy subjects. For decades, it was thought that the cerebellum was exclusively responsible for motor control [64]. Recently, assemblage evidence suggested that posterior cerebellum is involved in social cognition and perceiving and interpreting behavior, ranging from understanding concrete intentions, causes, emotions, and beliefs [65–67]. The previous study confirmed that cerebellar Crus2 is associated with late-onset and early-onset depression in adulthood [68]. Seeds from cerebellum regions conducted by connectivity analyses had been previously identified as the association of affective-limbic, executive, and motor networks.

The functional connectivity of individuals with depression decreased explicitly in affective-limbic and executive networks between several seed regions and increased between motor-related cerebellum seed regions [30]. Several reports indicated that the cerebellum altered neural response in depressed patients, including an increased blood flow of cerebellar vermis with cognitive impairment, decreased volume during a depressed state, and gradually decreased cerebellum over time [69, 70]. The previous studies suggested that cerebellum–ventromedial prefrontal cortex (vmPFC) may relate to cognitive function, while cerebellum–posterior cingulate cortex (PCC) relates to emotion processing [71]. Therefore, the cerebellum indicates abnormal activity in brain regions and relates to the reaction of depression. The cerebellum is essential as the field targeted approaches for treating depression [72]. Similar results also were authenticated in the cuneus [73]. In bipolar depression patients, gray matter volume in the cuneus is associated with better inhibitory control [74]. In addition, the primary function of the postcentral gyrus is somatosensory processing, which plays a vital role in somatic sensations, related to somatosensory responses [75].

Compared to the pretreatment, the ReHo values of right postcentral gyrus and insula were higher, and the left fusiform gyrus was lower in the posttreatment. MDD patients reduced responses significantly in left fusiform gyrus in facial emotion identification task [76, 77]. In several facial emotion processing studies, MDD adults exhibit brain activation differences in fusiform gyrus, and signal in left fusiform gyrus was associated with greater perceptual processing efficiency [78].

Although the results of HRSD-17 and neuroimaging confirmed that MECT is effective in MDD patients, several brain region signals remain extraordinary after treatment (Table 1, Supporting Information: Table S1). Compared to the healthy subjects, the values of left lingual gyrus, right calcarine cortex, and right mid occipital thalamus were higher in the posttreatment. Left calcarine cortex and right cerebrum were lower in the posttreatment than in the healthy subject. The calcarine cortex plays an essential role in refocusing attention [79]. The primary visual cortex of the calcarine cortex, located near the calcarine cortex, may send signals to the prefrontal cortex (PFC), which is located higher up in the brain. Guo et al. evidenced that the decreased values of voxel-mirrored homotopic connectivity represent an imaging biomarker for treatment-sensitive depression in the calcarine cortex [80]. These studies in fMRI resting state echo our conclusion above.

## 5. Limitation

Several limitations should be noted in the study. First, the sample size is not large enough. In data collection, COVID-19 broke out, and different related policies were released, such as quarantine for up to 7-14 days, resulting in the dilemma. Although we try to accumulate more samples from various websites and apps, the intensified difficulty still exists. Moreover, because of the limited samples, we did not provide placebo patient control (patients without MECT treatment). Second, we do not find the association between ReHo changes and HRSD-17 scores after MECT in the region difference (Supporting Information: Table S2). Besides HRSD-17, more indexes, such as Pittsburgh Sleep Quality Index (PSQI), Hamilton Rating Scale for Anxiety, Insomnia Severity Index (ISI), and Structured Clinical Interview for DSM-5 Disorders, should be taken to exclude the psychotic symptom. Some psychopathological features, including grandiosity, hostility, or excitement, prompt other dimensions of psychotic depression. Third, some cooccurring factors were lacking, such as smoking or substance use, essential in MDD patients. Fourth, the treatment of MDD is still hindered by the cognitive side effects of ECT. Research suggests that retrograde amnesia can persist in some patients following acute treatment for several months [61, 81, 82]. Modifying the pulse width significantly moderated retrograde memory outcomes for visual retrograde memory but not for auditory retrograde memory [60]. Therefore, future research, including cognition assessment over a standard treatment course, is warranted to determine whether ECT-related cognitive side effects exist. Finally, the clinical significance of ReHo requires further exploration. In the future, continuing follow-up will ensure their diagnosis with or without symptoms of psychosis.

## 6. Conclusion

The study confirmed that MECT improves psychotic symptoms in patients with first-episode major depressive disorder. The HRSD-17 and ReHo values identified brain regions that displayed differences between the pre- and posttreatment groups. Compared to the pretreatment, the ReHo values of the right postcentral gyrus and insula were higher, and the left fusiform gyrus was lower in the posttreatment. These results further contributed to a more tailored treatment approach to MDD from the pathophysiological and neuroimaging views.

## Figures and Tables

**Table 1:** Characteristics of the subjects.

Variables	Patients (*n* = 30)	HS (*n* = 35)	*p* values
Gender (M/F)	30 (8/22)	35 (10/25)	0.670
Age (mean ± SD)	14.71 ± 1.46	15.03 ± 2.07	0.415
HRSD-17 (mean ± SD)			
Pretreatment	28.84 ± 6.017		
Posttreatment	13.58 ± 8.804		<0.001⁣^∗^
BSSI (mean ± SD)			
Pretreatment	21.77 ± 5.920		
Posttreatment	8.03 ± 6.834		<0.001⁣^∗^

Note: ∗ indicates *p* values for paired *t*-tests. Abbreviations: SD: standard deviation; HRSD-17: 17­Item Hamilton Rating Scale for Depression; BSSI: Beck Scale for Suicidal Ideation.

**Table 2:** ReHo difference among pretreatment, posttreatment, and healthy subjects.

Brain areas (AAL)	Peak MNI coordinates			Cluster size	Peak *T* value
	*x*	*y*	*z*		
Pretreatment vs. control					
Bilateral cerebellum	3	-87	-36	47	3.2722
Cuneus-L	-9	-87	36	44	3.6084
Supramarginal-L	-57	-21	33	35	-3.7236
Postcentral-L	-24	-36	48	53	-3.4174
Posttreatment vs. pretreatment					
Insula-R	36	-18	6	24	3.9763
Postcentral-R	27	-24	45	22	3.7857
Fusiform-L	-42	-72	-18	21	-4.0763
Posttreatment vs. control					
Lingual-L	-27	-99	-18	20	3.3961
Calcarine-R	18	-102	-3	27	3.4308
Occipital-mid-R	33	-84	24	21	3.9381
Calcarine-L	-15	-69	12	15	-3.3871
Right cerebrum	21	-39	18	23	-4.0722

MNI: Montreal Neurological Institute; L: left; R: right.

## Data Availability

The data that support the findings of this study are available on request from the corresponding author. The data are not publicly available due to privacy or ethical restrictions.

## Supplementary Materials

Supplementary materials The association between ReHo changes and HRSD-17 scores before and after MECT in the region difference was explored, and the analysis results are shown in the supplementary material. However, these results were insignificant for multiple comparisons (*p* ≥ 0.05; see Table S1 and Table S2 for all results).
