# Supplementary material for: The Neuroimaging Role of Modified Electroconvulsive Therapy in the Major Depressive Disorder: Effectiveness in First-Episode Antipsychotic-Naive Major Depressive Disorder Patients
Source: Depress Anxiety. 2024 Feb 12;2024:9211145. doi: 10.1155/2024/9211145 (PMC11919022; doi:10.1155/2024/9211145)
Supplement: Supplementary materials — The association between ReHo changes and HRSD-17 scores before and after MECT in the region difference was explored, and the analysis results are shown in the supplementary material. However, these results were insignificant for multiple comparisons (p ≥ 0.05; see Table S1 and Table S2 for all results). [file 9211145.f1.docx]

Supplemental Material

For

**The neuroimaging role of modified electroconvulsive therapy in the major depressive disorder: Effectiveness in first-episode, antipsychotic-naive major depressive disorder patients**

**Table S1 Association between ReHo value and HRSD-17 scores in patient before MECT**

| Brain areas (AAL) | | Unstandardized Coefficients | | Standardized Coefficients | Sig. |
| --- | --- | --- | --- | --- | --- |
|  |  | B | Std. Error | Beta |  |
|  | Bilateral_Cerebelum | -10.87 | 5.32 | -0.45 | 0.05 |
|  | Cuneus_L | -4.43 | 13.24 | -0.06 | 0.74 |
|  | SupraMarginal_L | 11.21 | 18.56 | 0.21 | 0.55 |
|  | Postcentral_L | -4.73 | 14.50 | -0.11 | 0.74 |

Note: Variable were analyzed in a linear mixed model controlling for age, gender, and scanner site(p < .05).

**Table S2 Association between ReHo changes and HRSD-17 scores after MECT**

| Brain areas (AAL) | | Unstandardized Coefficients | | Standardized Coefficients | Sig. |
| --- | --- | --- | --- | --- | --- |
|  |  | B | Std. Error | Beta |  |
|  | Fusiform_L | -2.51 | 22.71 | -0.02 | 0.91 |
|  | Insula_R | 25.14 | 20.28 | 0.24 | 0.22 |
|  | Postcentral_R | 13.14 | 16.32 | 0.15 | 0.42 |

Note: Variable were analyzed in a linear mixed model controlling for age, gender, and scanner site(p < .05).
